# Mechanisms Underlying Decision-Making as Revealed by Deep-Brain Stimulation in Patients with Parkinson’s Disease

**DOI:** 10.1016/j.cub.2018.02.057

**Published:** 2018-04-23

**Authors:** Damian M. Herz, Simon Little, David J. Pedrosa, Gerd Tinkhauser, Binith Cheeran, Tom Foltynie, Rafal Bogacz, Peter Brown

**Affiliations:** 1 MRC Brain Network Dynamics Unit at the University of Oxford, Mansfield Road, Oxford OX1 3TH, UK; 2 Nuffield Department of Clinical Neurosciences, University of Oxford, Level 6, West Wing, John Radcliffe Hospital, Oxford OX3 9DU, UK; 3 Sobell Department of Motor Neuroscience and Movement Disorders, University College London Institute of Neurology, 33 Queen Square, London WC1N 3BG, UK; 4 Department of Neurology, University Hospital Marburg, Baldingerstrasse, 35043 Marburg, Germany; 5 Department of Neurology, Bern University Hospital, Freiburgstrasse, 3010 Bern, Switzerland

**Keywords:** decision threshold, decision-making, drift diffusion model, subthalamic nucleus, Parkinson’s disease, deep-brain stimulation, beta

## Abstract

To optimally balance opposing demands of speed and accuracy during decision-making, we must flexibly adapt how much evidence we require before making a choice. Such adjustments in decision thresholds have been linked to the subthalamic nucleus (STN), and therapeutic STN deep-brain stimulation (DBS) has been shown to interfere with this function. Here, we performed continuous as well as closed-loop DBS of the STN while Parkinson’s disease patients performed a perceptual decision-making task. Closed-loop STN DBS allowed temporally patterned STN stimulation and simultaneous recordings of STN activity. This revealed that DBS only affected patients’ ability to adjust decision thresholds if applied in a specific temporally confined time window during deliberation. Only stimulation in that window diminished the normal slowing of response times that occurred on difficult trials when DBS was turned off. Furthermore, DBS eliminated a relative, time-specific increase in STN beta oscillations and compromised its functional relationship with trial-by-trial adjustments in decision thresholds. Together, these results provide causal evidence that the STN is involved in adjusting decision thresholds in distinct, time-limited processing windows during deliberation.

## Highlights

• We performed temporally patterned stimulation of the subthalamic nucleus in humans
• During stimulation, Parkinson’s patients performed a perceptual decision-making task
• Stimulation effects on behavior were confined to a short window during deliberation
• Here, stimulation affected changes in decision thresholds during difficult decisions

## Teaser

In this article, Herz et al. provide causal evidence that the subthalamic nucleus mediates adjustments of decision thresholds, depending on the difficulty of the decision. This adjustment is processed during a short time window early during deliberation and is related to changes in oscillatory activity of the subthalamic nucleus.

## Introduction

In everyday decisions, we need to determine how much evidence we wish to collect before committing to a choice. For example, dwelling over which meal to pick during a lunch break might make us miss out on valuable time that we could spend chatting with our friends, whereas quickly choosing a menu option without proper thought might make us overlook a better alternative. In a modeling framework, optimizing this trade-off between the speed and accuracy with which we make decisions can be implemented through a decision threshold that specifies the amount of evidence that is required for making a choice [1, 2, 3]. It has been inferred from behavioral data that humans may adjust their decision threshold on the basis of both the instruction to be fast or accurate given before a task [4, 5] and the difficulty of the decision to be made as the task unfolds [6, 7, 8]. The process of adjusting the decision threshold according to task difficulty was recently investigated in a behavioral study, which suggested that humans determine the difficulty of the current decision after a brief period of integrating evidence, and only then adjust the decision threshold in a single abrupt change [6].

Converging evidence from computational, electrophysiological, and neuroimaging studies points to a pivotal role of the subthalamic nucleus (STN) in adjustments of such a decision threshold [9, 10, 11, 12, 13, 14]. For example, a study recording STN activity during decision-making reported that the amplitude of beta oscillations (13–30 Hz) changed according to instructions early into a given task (150–400 ms after stimulus onset) and according to task difficulty later during the task (after ∼500 ms) [12]. This has received further support from behavioral studies in Parkinson’s disease (PD) patients who are treated with STN deep-brain stimulation (DBS), a highly effective treatment for PD and other neurological disorders [15, 16]. While alleviating motor dysfunction in PD, STN DBS has been shown to decrease the time that patients take for making difficult decisions, sometimes resulting in suboptimal choices [17, 18, 19]. However, the mechanisms underlying these behavioral observations remain elusive. One hypothesis is that DBS reduces the effective decision threshold on difficult trials by removing the “braking signal” that the STN applies throughout the decision process. An alternative hypothesis is that STN DBS only interferes with the mechanism setting the decision threshold to the required level. These two hypotheses make different predictions on the window in which DBS should have an effect: the first hypothesis predicts an effect of DBS around the time of choice (when the decision threshold is reached), whereas the second hypothesis predicts an earlier effect (when the decision threshold is set).

**Figure 1:**
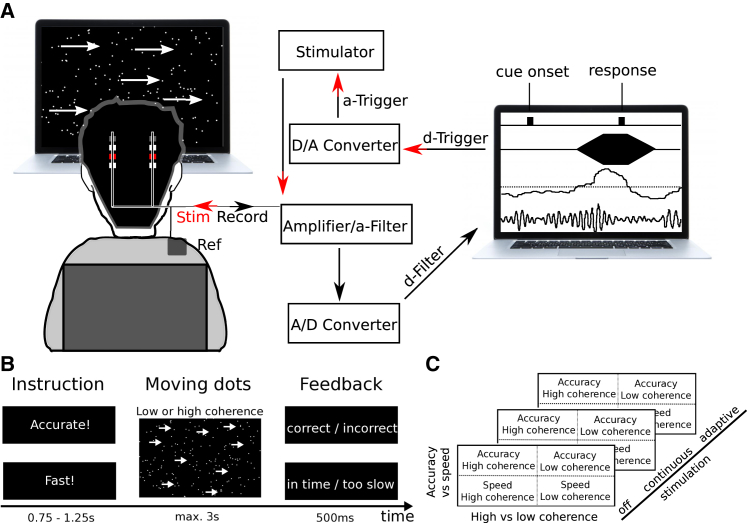
Experimental Setup and Task (A) During the experiment, bipolar local field potential (LFP) recordings were pre-processed (analog 3–37 Hz filter; amplification with common-mode rejection; analog-to-digital conversion) and analyzed online (digital filter around individual beta peak; rectification and moving averaging) to obtain a continuous measure of beta activity. Whenever beta activity crossed a pre-defined threshold (median of beta power), stimulation was triggered. Pseudo-monopolar 130 Hz DBS was ramped up for 250 ms and ramped off again when beta fell below the threshold. Task events (moving dots cue onset and responses) were recorded in the same software (Spike2) that recorded LFPs and controlled stimulation. (B) The task design comprised speed versus accuracy instructions (effect of instruction) and moving dots cues with either low (8%) or high (50%) coherence (effect of coherence). (C) All patients performed the task three times; off DBS; with continuous DBS; and with adaptive DBS, where stimulation was trigged by beta activity (see above). See also Figures S1 and S5 and Table S1.

The aim of this study is to probe the mechanisms by which the STN influences decision making, as revealed by DBS in patients with PD. We applied DBS in specific times during decision making, which allowed us to distinguish between the alternative mechanisms of DBS described above and to assess their neural correlates. We assessed 10 PD patients who performed a perceptual decision-making task in the immediate postoperative period after STN DBS surgery in three separate sessions: off DBS, with continuous DBS (cDBS), and with adaptive DBS (aDBS). Seven patients completed the study. During aDBS, 130 Hz stimulation was only turned on when simultaneously recorded STN beta activity exceeded a threshold defined by the median beta power of each individual patient and turned off again as soon as beta activity fell below that threshold. This has been shown to improve motor function in PD to at least a similar extent as “conventional” cDBS [20] and to abort dynamic elevations in beta oscillatory activity [21], despite the fact that aDBS delivers stimulation <50% of the time (see Figures 1A and S1A).

## Results

In addition to the large stun effect (53% on average), which reflects the temporary clinical improvement after DBS electrode insertion without applying stimulation or intake of dopaminergic medication, we found that DBS alleviated motor symptoms on average by a further 22% (*Z* = −2.35; p = 0.019) compared to off DBS. This clinical effect did not differ between cDBS and aDBS (median improvement during cDBS was 22.2% versus 22.9% during aDBS; *Z* = −0.73; p = 0.463).

### Dynamic Effects of STN DBS on Decision-Making

**Figure 2:**
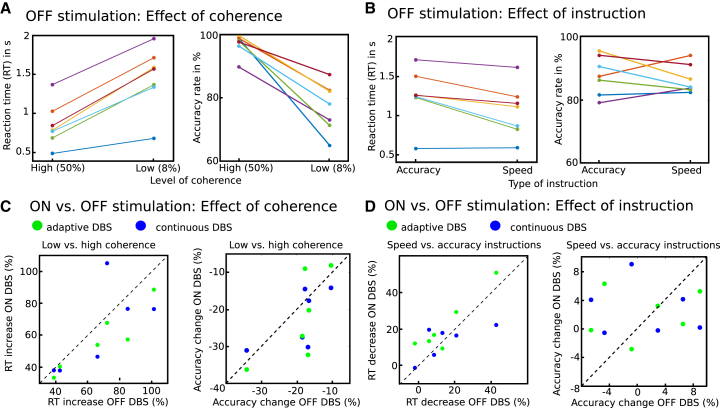
Behavioral Results (A) Effects of coherence on RT and accuracy rates are shown for each individual patient (n = 7). (B) Same as (A) for effects of instruction. (C) Effects of coherence and instruction on RT and accuracy during stimulation (adaptive and continuous DBS) versus off DBS for each patient (n = 6 in 2 different stimulation regimes). (D) Same as (C) for effects of instruction.

During the task, patients had to decide whether a cloud of moving dots appeared to move to the left or to the right on a computer screen. The percentage of dots moving coherently to one direction was either high (50%) or low (8%), and patients were instructed to respond as fast or as accurately as possible. Thus, patients had to adapt to differing levels of difficulty (effect of coherence) and to explicit task instructions (Figures 1B and 1C). Without stimulation (off DBS), patients responded significantly slower during low- compared to high-coherence trials (median difference: 677 ms; *Z* = 2.37; p = 0.018) and responded faster when instructed to weight speed over accuracy (median difference: 149 ms; *Z* = 2.2; p = 0.028; see Figures 2A and 2B). The effect of coherence did not significantly differ depending on speed versus accuracy instruction and vice versa (*Z* = 0.315; p = 0.753). Accuracy rates were lower during low- compared to high-coherence trials (78% versus 98%; *Z* = 2.37; p = 0.018) but were not significantly different between speed and accuracy instructions (88% after accuracy versus 84% after speed instruction; *Z* = 0.51; p = 0.612; see Figures 2A and 2B). Together, these results are in line with a previous study testing a separate group of PD patients and healthy participants using the identical task [12].

Applying DBS diminished the extent to which participants slowed down responses depending on task difficulty, i.e., it reduced the response time (RT) difference between low- and high-coherence trials, on average by 7% (*Z* = −2.12; p = 0.034), and this effect did not differ depending on the types of stimulation (median reduction in slowing down during cDBS was 6.5% versus 8.8% during aDBS; *Z* = 0.52; p = 0.6; see Figure 2C). Conversely, DBS did not significantly affect the extent to which patients reduced RT after speed versus accuracy instructions (during DBS, patients showed on average 5.9% more RT reduction after speed instructions, *Z* = 1.41, p = 0.158; change during cDBS −1.1% versus 8.1% during aDBS, *Z* = −1.57, p = 0.116; see Figure 2D). There were no effects of DBS on accuracy rates (coherence: effect of stimulation *Z* = 1.41, p = 0.158; cDBS versus aDBS *Z* = 0.32, p = 0.753; instruction: effect of stimulation *Z* = 0.71, p = 0.48; cDBS versus aDBS *Z* = 0.1, p = 0.917; see Figures 2C and 2D). In summary, the behavioral results show that DBS affected the increases in RT due to difficult stimuli.

**Figure 3:**
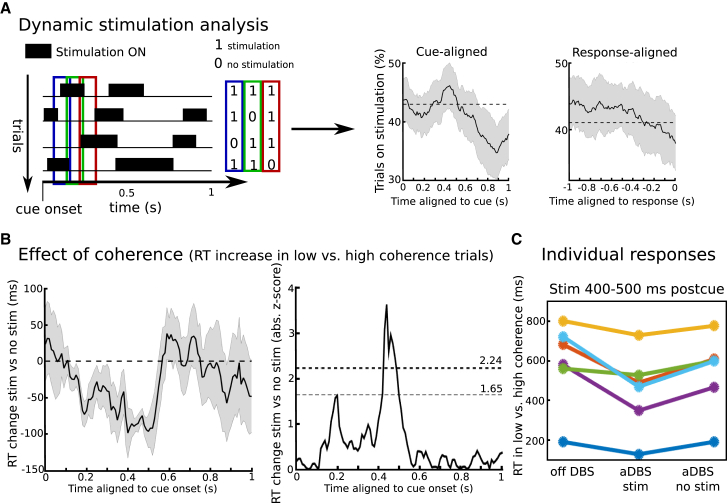
Analysis of Stimulation Patterns during aDBS (A) During aDBS, stimulation was turned on and off depending on STN beta activity. Therefore, stimulation was applied at different time points in different trials. For 100-ms-long time windows, which were shifted relative to cue onset and response, respectively, trials were marked as “stimulation” and “no stimulation”. This is shown schematically for three consecutive time windows (blue, green, and red rectangles). The number of trials in which stimulation was applied varied depending on task-related changes in beta power (see STAR Methods), but throughout all time windows, the % trials with stimulation turned on ranged between 35% and 45% on average (right panel). Dotted lines indicate median % trials on stimulation over time. (B) Stimulation significantly decreased the extent to which participants slowed down responses depending on task difficulty (negative values indicate that the effect of coherence was lower during stimulation versus no stimulation) in a distinct time window 400–500 ms postcue, but not in other time windows. Mean ± SEM in ms is shown in the left panel and absolute *Z* scores (mean/SD) in the right panel. Note that the effect of stimulation in each given time window is compared against trials in which stimulation could occur in any other time window. The statistical threshold for each time window was set to *Z* = 2.24 to correct for the four separate tests, and correction for multiple time windows was conducted using cluster-based permutation tests. An uncorrected threshold of *Z* = 1.65 is shown for illustration purposes. Shaded areas represent SEM. (C) Changes in effect of coherence during 400–500 ms postcue stimulation shown for individual patients. For remaining tests (effect of instruction and response-aligned windows), see Figure S3. See also Figure S2 and Table S2.

Using aDBS allowed us to determine in which temporal window stimulation might have an effect. During aDBS, stimulation was triggered by STN beta power, i.e., DBS was turned on when beta power was high and turned off when beta power was low (Figure S1A). Due to variations in beta power over time and between trials (Figure S1B), stimulation during aDBS was applied at different time windows in different trials. In a first step, we assessed whether stimulation was applied or not in each trial over dynamically shifting time windows. This showed that, after onset of the moving dots, cue stimulation was applied on ∼45% of trials on average until about 700 ms postcue, when it decreased to ∼35% (Figure 3A). We then compared RT for stimulation and no-stimulation trials in each time window and tested whether stimulation altered the extent to which patients slowed down depending on task difficulty (effect of coherence) and explicit instructions. This was computed for windows relative to cue onset and motor response, respectively, and corrected for the high number of statistical tests using cluster-based permutation tests (see STAR Methods for more details). Analysis of moving windows aligned to the moving dots cue revealed a highly significant effect of stimulation on the extent to which participants slowed down their responses according to task difficulty but only in a specific time window ∼400–500 ms after cue onset (Figure 3B). This effect remained stable with the use of different moving windows (of 50 ms, 100 ms, and 200 ms; Figure S2). Conversely, we did not find any significant effects of cue-locked stimulation on RT differences between speed and accuracy instructions or any effects of response-locked stimulation (Figure S3). Furthermore, the observed effect of cue-locked stimulation 400–500 ms postcue was significantly stronger than that of response-locked stimulation (from 500 ms prior to responding until the response) when compared directly against each other (*Z* = 2.028; p = 0.043; Wilcoxon signed rank test). In summary, the effect of DBS on the speed of difficult decisions was confined to a remarkably brief period during the deliberation process.

Because stimulation during aDBS was triggered by beta power, it is possible that the observed stimulation effects on patients’ ability to slow down were driven by trial-wise fluctuations in beta power rather than stimulation per se. To control for this possible confound, we repeated the same time window analyses as above using “surrogate stimulation” off DBS as a control condition, which we computed offline by assessing when stimulation theoretically would have been triggered in the off DBS condition (see STAR Methods and Figure S1C for more details). Both real stimulation during aDBS and surrogate stimulation off DBS were closely related to temporal changes in STN beta power, but only during aDBS was real stimulation applied. We did not find any behavioral effects of surrogate stimulation in the time windows analysis (effect of coherence during surrogate stimulation 400–500 ms postcue was on average −20 ms, *Z* = 0, and p = 1, which was significantly weaker than the effect of real stimulation 400–500 ms postcue, *Z* = 2.37 and p = 0.018, Wilcoxon signed rank tests), showing that the timing-specific behavioral effects during aDBS were due to stimulation, not changes in beta power. Furthermore, there were no differences in the % of trials in which stimulation was applied between low- and high-coherence trials during aDBS 400–500 ms postcue (44% versus 48%; *Z* = 1.18; p = 0.237; Wilcoxon signed rank test), indicating that the behavioral effect was not related to changes in the likelihood of aDBS being applied in trials with different coherence.

### DBS Alters Dynamic Modulations of Decision Thresholds

**Figure 4:**
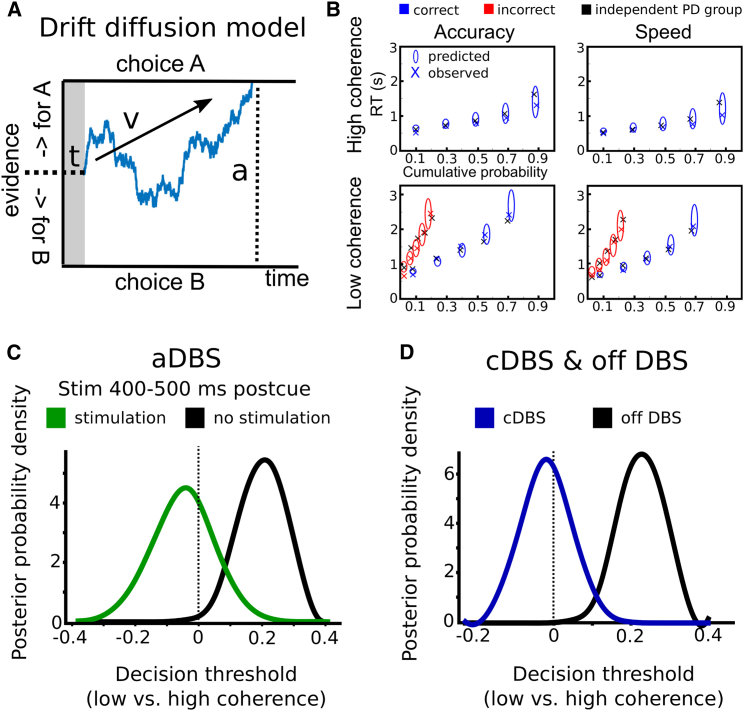
Effects of DBS on Decision Making Parameters (A) Schematic illustration of the drift diffusion model. Evidence for choice A versus choice B is accumulated over time until it reaches either boundary for choice A or B. When the boundary is reached, the respective choice is selected. The slope of the accumulated evidence depends on drift rate *v*. The distance between the two boundaries is determined by decision threshold *a*. The non-decision time *t* is related to afferent delay, sensory processing, and motor execution. The blue trace schematically represents a trial in which choice A wins over choice B. (B) Quantile probability plots showing the observed (x) and predicted (ellipses) RT against their cumulative probabilities (10, 30, 50, 70, and 90 percentiles × accuracy rates). The widths of the ellipses represent uncertainty (SD of the posterior predictive distribution). Blue symbols are used for correct and red symbols for incorrect trials. Note that predictions of accuracy can be inferred from the horizontal alignment of ellipses (predictions) and crosses (observed data). The black crosses represent RT from an independent patient group (n = 11) [12] that was not used for model fitting and shows the generalizability of the model predictions. (C) Posterior probability densities of model parameters for changes in decision thresholds in low- versus high-coherence trials during aDBS. Decision thresholds increased in low- versus high-coherence trials if stimulation was not applied 400–500 ms postcue, but this effect was absent when stimulation was applied in this time window. (D) Posterior probability densities of model parameters for changes in decision thresholds in low- versus high-coherence trials for cDBS and off DBS.

How can STN DBS affect patients’ behavior during decision making? One possibility is that stimulation interferes with (physiological) adjustments of decision thresholds, which have been related to modulations of STN activity during deliberation [11, 12, 13, 14]. However, gross measures of task performance, such as response times, alone cannot disentangle the different mechanisms underlying decision-making. Therefore, we computed the latent decision-making processes underlying the observed behavior using drift diffusion modeling (DDM). During perceptual decision making, sensory information (here, direction of moving dots) has to be transformed into a categorical choice (here, right versus left button press). In the DDM framework, this process is characterized by the accumulation of noisy evidence with drift rate *v* until the accumulated evidence reaches a boundary or decision threshold *a* (see Figure 4A). Whereas the drift rate is mainly determined by the sensory cue (high-coherence trials are thought to have a high drift rate compared to low-coherence trials, resulting in fast and accurate decisions), the decision threshold determines how cautiously people respond, i.e., how much evidence they require before committing to a choice. Finally, the model has a parameter *t*, the non-decision time, which reflects all processes not directly related to deliberation, such as afferent delay, early sensory processing, and motor execution. In a first step, we modeled the latent decision-making parameters underlying the observed task-related changes in behavior without stimulation (off DBS) using Bayesian hierarchical drift diffusion modeling (HDDM) [22]. We used a simple *a priori*-defined model, which was fitted to the observed behavior. The drift rate was allowed to vary between low- and high-coherence trials and the decision threshold between speed and accuracy instructions [12]. Based on ideal observer models and empirical evidence [7, 8, 23], we also allowed thresholds to vary between trials with low and high coherence. Importantly, posterior predictive checks (QP plots; see STAR Methods) showed that this simple model predicted the observed behavior well (Figure 4B) and even closely predicted the behavior from our previous study in an independent patient group with 11 subjects [12], which was not used for model fitting (black crosses in Figure 4B). Assessment of model parameters showed that drift rates were lower in low- compared to high-coherence trials (100% posterior probability) and decision thresholds were lower after speed compared to accuracy instructions (100% posterior probability). Furthermore, decision thresholds were also modulated by task difficulty with higher thresholds in low- compared to high-coherence trials (100% posterior probability).

Given that changes in drift rates, decision thresholds, and non-decision times all affect response times, the observed timing-specific effects of stimulation on patients’ ability to slow down could be related to any of these mechanisms. To disentangle effects of stimulation on distinct decision-making processes, we fitted the model to the observed behavior during aDBS, marking each trial as “stimulation” if, in this given trial, stimulation was applied 400–500 ms postcue and “no stimulation” if DBS had not been delivered in this time window. We allowed stimulation to alter drift rates, decision thresholds, and non-decision times and inspected the posterior parameter distribution to assess significant changes of stimulation on model parameters (see STAR Methods). This revealed that decision thresholds were affected by cue-locked stimulation, depending on the type of coherence (98% probability for stimulation × coherence interaction), but not depending on instructions (78% probability for stimulation × instruction interaction), nor was there a significant main effect of stimulation (93% probability). Furthermore, there were no effects of stimulation on drift rates or non-decision times (all probabilities <80%). Post hoc tests showed that decision thresholds were higher in low- versus high-coherence trials if no stimulation was applied 400–500 ms after the cue (>99.5% probability), but this effect was absent when stimulation was applied in this time window (33% probability for thresholds in low coherence > high coherence; see Figure 4C). Furthermore, including the stimulation × coherence interaction improved model evidence compared to a model without this interaction (deviance information criterion [DIC] 1,906 versus 1,910; lower values indicating stronger evidence). This was only the case for cue-locked stimulation 400–500 ms postcue, whereas including a stimulation × coherence interaction for response-locked stimulation prior to the response (see above) did not improve model evidence (DIC 1,910) nor was the interaction with coherence significant for response-locked stimulation (69% probability). Thus, during aDBS, both task performance and task-related adjustments in decision-making parameters were highly similar to off DBS as long as stimulation did not fall into the time window 400–500 ms after the moving dots cue, whereas—if stimulation was applied in this time period—patients’ ability to slow down responses in difficult trials was diminished in tandem with an abolished difficulty-related increase in decision thresholds (see Figures 3C, 4C, and 4D). Finally, we analyzed whether decision thresholds were modulated according to task difficulty (coherence) during cDBS by conducting the same HDDM analysis as off DBS, including the behavioral data recorded during cDBS in the model. This showed a significant interaction between stimulation (cDBS) and no stimulation (>99.9% probability), because thresholds were only higher in low- compared to high-coherence trials off DBS (100% probability), but not during cDBS (42% probability for thresholds in low coherence > high coherence; Figure 4D).

### Neural Correlates of DBS-Induced Changes in Decision Making

**Figure 5:**
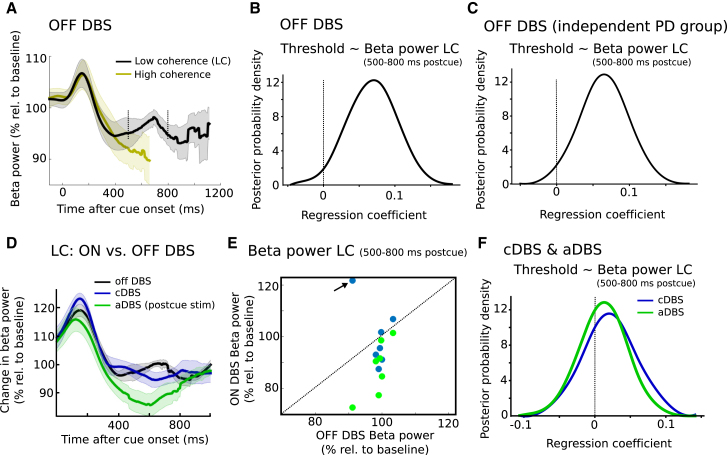
Changes in STN Activity (A) Changes in beta power after cue onset off DBS shown for low (black trace) and high (yellow trace) coherence separately. Traces are plotted until median RT of the respective conditions. Shaded areas represent SEM. (B) Beta power 500–800 ms postcue during low coherence trials off DBS predicts increased decision thresholds at the single-trial level. (C) Same as (B) for an independent patient group (n = 11) [12]. (D) Change in beta power from 0 to 1 s postcue for off DBS, cDBS, and aDBS (400–500 ms postcue stimulation). All conditions are matched for fluctuations in beta power (see STAR Methods for more details). Data from an outlier in cDBS are omitted from this plot but do not affect statistical results (see main text). Shaded areas represent SEM. (E) Changes in 500–800 ms beta power postcue for all individual patients during cDBS and aDBS (400–500 ms postcue stimulation). Data from the outlier omitted in (D) are arrowed. (F) Same as (B) and (C) but for cDBS and aDBS. The relationship between beta power 500–800 ms postcue and trial-by-trial adjustments in decision thresholds that was observed off DBS is absent in cDBS and aDBS. Note that, in (B), (C), and (F), non-standardized regression coefficients are shown. See also Figure S4.

Previous studies have demonstrated that decision threshold adjustments are reflected by changes in STN activity using implanted DBS electrodes for LFP recordings [12, 13]. The custom-built DBS device [24] used in the current study allowed us to record STN beta activity not only when DBS was turned off but also during stimulation (see STAR Methods) in order to assess the effects of stimulation on STN activity. We first verified that STN beta power was modulated according to task instructions. Beta power decreased early after cue onset (∼150–400 ms postcue) to a similar extent as observed in previous studies [12, 25, 26]. Replicating the results from our previous study [12], we found that this cue-induced decrease in beta power was steeper after speed compared to accuracy instructions (*Z* = 2.2; p = 0.028), but not different between low- and high-coherence trials (*Z* = 0.169; p = 0.866), and predicted decreased decision thresholds at the single-trial level (96% probability; see Figure S4). This relationship between STN beta power and decision thresholds can, however, not explain the observed behavioral effects of STN stimulation on patients’ ability to slow down, which occurred later (400–500 ms postcue) and depended on dots coherence not task instructions. Thus, we plotted changes in STN beta power after the cue separately for low- and high-coherence trials (Figure 5A). Here, differences in beta power became apparent starting ∼500 ms after cue onset, at which point beta further decreased in high-coherence trials and showed a relative increase in low-coherence trials lasting until ∼800 ms. Because stimulation during aDBS lagged behind changes in STN beta power (see STAR Methods), the difference in beta from 500 to 800 ms postcue between low- and high-coherence trials did not lead to differences in the likelihood of aDBS being applied in the same time window (stimulation was turned on in 40% of trials in this time period both for low- and high-coherence trials; *Z* = 0; p = 1; Wilcoxon signed rank test). Also of note, beta power in low-coherence trials 500–800 ms postcue was only very weakly, and not significantly, correlated with the early (150–400 ms) cue-induced beta decrease (Spearman correlation was significant in 1/7 patients; average rho = −0.1; p = 0.078; Wilcoxon test on r to z transformed within subject correlation coefficients). However, differences in beta power between low- and high-coherence trials might simply reflect the strong RT difference, because beta power is known to decrease during the motor response [12]. Thus, to more directly test whether the observed relative beta increase during low-coherence trials was related to changes in decision thresholds, we entered single-trial estimates of beta power from 500 to 800 ms postcue during low-coherence trials in the HDDM regression analysis (see STAR Methods for more details). This revealed a positive relationship between beta power and decision thresholds (98% probability), i.e., high beta power between 500 and 800 ms predicted increased thresholds (Figure 5B). This remained significant even when excluding trials with RT < 800 ms and was reproducible in an independent PD group of 11 subjects [12] (99% probability; Figure 5C).

How were these changes in beta power and their relationship with decision threshold adjustments affected by DBS, which has previously been shown to suppress STN beta power [27, 28, 29]? To investigate this, we analyzed STN activity during low-coherence trials during aDBS (400–500 ms postcue stimulation), cDBS, and off DBS. Stimulation abolished the relative increase in beta power during low-coherence trials in the 500–800 ms postcue period (*Z* = 1.98; p = 0.048). This effect was driven by cue-locked stimulation during aDBS, which decreased beta power in this time period more strongly than cDBS (*Z* = 2.37; p = 0.018; see Figures 5D and 5E). Both statistical tests also remained significant when excluding an outlier value (see Figure 5E; *Z* = 2.69, p = 0.007 for DBS versus off and *Z* = 2.2, p = 0.028 for aDBS versus cDBS). Given that stimulation reduced STN beta power in a time period where beta power normally, i.e., off DBS, correlated with modulations of decision thresholds, stimulation might compromise the relationship between changes in STN activity and threshold adjustments. To test this, we entered single-trial estimates of STN beta power 500–800 ms postcue during aDBS and cDBS into the HDDM analogously to the HDDM regression analysis off DBS. Both cDBS and aDBS abolished the relationship between threshold adjustments and STN beta power, with the most likely regression coefficient (mean of the posterior distribution) being close to 0 (0.02 for cDBS and 0.01 for aDBS; 73% and 64% probability for regression coefficient >0, respectively; see Figure 5F). Thus, stimulation did not only affect the power of STN beta activity but also its relationship with trial-by-trial modulations of decision thresholds.

## Discussion

When making decisions, we do not only have to decide what to choose but also when to choose it. Like Buridan’s donkey being stuck midway between an equally appealing stack of hay and bucket of water, we must decide how much time we can spend on deliberation before committing to a choice. Importantly, in ecologically realistic situations, decision thresholds have to be flexibly adapted to changing environments. For example, if we realize that the value of two options is nearly identical, the optimal decision-making policy is to decrease decision thresholds and to pick one of the options, even if their relative difference is close to zero [7, 23, 30, 31]. Conversely, in other scenarios, e.g., making sequential decisions with differing (low-to-medium) difficulty, it might be advantageous to increase our thresholds once we realize that the current decision is relatively difficult as suggested by ideal observer models and empirical evidence [7, 8, 23]. Similar to the latter scenario, in the current task, we found that patients had relatively increased decision thresholds in difficult compared to easy trials as well as lower decision thresholds after instructions emphasizing speed over accuracy. We show that DBS affected patients’ ability to adjust decision thresholds according to task difficulty, but *only* if applied in a specific temporally confined time window during deliberation. Stimulation 400–500 ms after onset of the moving dots cue alone diminished slowing of response times that occurred on difficult trials off DBS. Furthermore, it eliminated a relative, time-specific increase in STN beta oscillations and compromised its functional relationship with trial-by-trial adjustments in decision thresholds, implicating this time-specific beta modulation in the process of decision threshold adjustment according to task difficulty.

Remarkably, DBS did not influence RT when applied later than 500 ms after stimulus onset, despite the fact that the vast majority of responses during difficult trials (>99%) were made later than 500 ms. This argues against the idea that DBS reduces the elevation of STN activity during difficult trials removing a braking signal that the STN exerts throughout the decision process. Instead, we found that DBS reduced the dependence of RT on difficulty only when applied in a brief (∼100 ms) window. This implies that DBS interfered with a time-limited process of setting the decision threshold to the required level according to task difficulty. As mentioned earlier, it has been recently proposed that decision threshold is set according to task difficulty in a single abrupt change depending on information gathered in an initial period [6], which is in line with a computational model developed by Frank and colleagues [8, 10]. Our results identify a potential neural correlate of this process and raise the possibility that it is dependent on the STN. The time-limited effect of stimulation we observed is consistent with optogenetic studies in rodents [32, 33] showing that behavioral effects of stimulation are only observed for specific “critical” time windows during deliberation.

It is likely that other factors determining adjustments in decision threshold also involve changes in STN activity that take place in relatively brief, discrete, and context-determined time windows. Indeed, the results of the present study taken together with those of a previous study using the same paradigm off DBS [12] reveal three distinct mechanisms adapting decision threshold, whose signatures are visible in the local field potential (LFP) recorded in STN. First, beta power showed a consistent decrease from ∼150 to 400 ms after onset of the moving dots cue, and in line with a previous study [12], this was modulated by task instructions with a steeper decrease after speed compared to accuracy instructions and correlated with trial-by-trial variations in decision thresholds. Second, ∼500 ms postcue, beta power further decreased in easy trials but showed a relative increase in difficult trials. Single-trial variations in beta power during this relative increase ∼500–800 ms after the cue did not correlate with the earlier (150–400 ms) cue-induced decrease in beta power but were significantly correlated with trial-by-trial variations in decision thresholds. Third, the previous study [12] also reported that activity in 2–8 Hz oscillations (which we were not able to record using the closed-loop device applied in the current study) differed after speed and accuracy instructions. This difference started ∼500 ms after stimulus onset, and similar to another study [13], 2–8 Hz power only correlated with decision threshold when patients responded with caution, i.e., after accuracy instructions. Furthermore, there was no correlation between instruction-related changes in STN beta and 2–8 Hz power. Changes in theta power have also been related to decision threshold adjustments at the cortical level. Cavanagh et al. [17] found correlations between trial-by-trial adjustments in decision thresholds and single-trial recordings of theta power recorded over prefrontal cortex in healthy people and patients with Parkinson’s disease. Interestingly, this relationship was inverted when STN DBS was applied. In addition, a recent study indicated that low-frequency (4 Hz) STN DBS might improve patients’ ability to adjust decision thresholds in an interval-timing task [34]. Together, these data suggest that distinct changes in STN activity and interconnected cortical areas underscore dynamic within-trial changes in decision thresholds. The fact that proactive changes in decision threshold engendered by speed over accuracy instruction may involve more than one processing window [12] might also explain why DBS did not significantly abolish instruction-related RT effects in the present study.

The extent to which the changes in STN activity reflected modulations of “decision-related” prefrontal STN and movement-related motor STN computations remains to be clarified. Because of current spread around the stimulated electrode, we do not believe that DBS in the current study was necessarily only delivered to the motor subregion of the STN, even though the contact for stimulation was based on the strongest expression of beta oscillations, which have been primarily related to the “motor” areas of the STN [35]. Rather, there are several indications that, in the current study, DBS interfered with processes related to deliberation rather than motor processing, consistent with the spread of stimulation to adjacent “cognitive” or “associative” parts of the STN. First, the critical effect of DBS occurred 400–500 ms after the moving dots cue, which is ∼1 s before the average motor response in low-coherence trials. Second, there were no behavioral effects of stimulation when aligned to the response, which would be expected for effects of DBS on movement preparation per se.

LFPs are thought to represent coordinated synaptic input [36, 37]. If correct, then this implies that changes in beta synchronization are being imposed by afferents to the STN, so that the STN, together with its efferent connections, may help implement the change in threshold rather than decide it. Our results pertain to patients with PD, and the extent to which they generalize to the healthy state remains to be clarified. Given its invasive nature, we were not able to apply DBS in a healthy control group. However, a previous study using the identical task has shown that differences in instructions and coherence levels lead to similar changes in task performance as well as latent decision-making processes in PD patients and healthy people [12]. Another shortcoming of our study is the limited sample size and trial count, and for this reason, we did not conduct extensive model comparisons comprising all possible interactions between task manipulations, DBS, and model parameters. Further studies with larger sample sizes and detailed analyses of movement kinematics are warranted in this regard.

Together, we demonstrate and define a causally important time window of STN involvement in the process of decision making. Together with modulations in other frequency bands, in particular low-frequency/theta power [11, 12, 13, 17], and activity changes in cortical areas [11, 12, 13, 17, 38, 39, 40], our observations add to the converging evidence that decision thresholds are adjusted through dynamic modulations of cortico-basal ganglia networks.

## STAR★Methods

### Key Resources Table

**Table:** 

REAGENT or RESOURCE	SOURCE	IDENTIFIER
**Deposited Data**		

Behavioral and neurophysiological data related to the project	This paper	https://ora.ox.ac.uk/objects/uuid:0cdf0eda-3a1d-4b66-8edd-0f51d824f6cc

**Software and Algorithms**		

PsychoPy v1.8	[45] http://psychopy.org/installation.html	RRID:SCR_006571
MATLAB R2015a	https://se.mathworks.com/products/matlab.html	RRID:SCR_001622
HDDM implemented in python 2.7	[22] http://ski.clps.brown.edu/hddm_docs/	RRID:SCR_ 008394

### Contact for Reagent and Resource Sharing

Further information and requests for resources and data should be directed to and will be fulfilled by the Lead Contact, Prof. Peter Brown (peter.brown@ndcn.ox.ac.uk).

### Experimental Model and Subject Details

#### Participants

The current study was conducted in Parkinson’s disease (PD) patients in the immediate post-operative period after DBS surgery of the bilateral subthalamic nucleus (STN). Between September 2015 and August 2017, ten patients (8 males, average age 57 years) were enrolled in the study (Table S1). Three of the included patients were not able to perform the task due to fatigue and had to be excluded. Thus, seven patients completed the experiment 2–7 days after electrode implantation. As described below this relatively small sample size was estimated to be sufficient given the scale and variance of predicted effects. In addition, we were careful to collect a high number of experimental trials (720 trials per patient resulting in 5040 trials combined for the single trial analyses, see below), and we also validated our findings in archival cohorts where possible (see results). All patients were right-handed as revealed by the Edinburgh-Handedness Inventory [41]. Lead localization was verified by stereotactic intraoperative magnetic resonance imaging (London) or by monitoring the clinical effect and side effects of test stimulation during operation and immediate postoperative stereotactic computerized topography (Oxford). During the experiment local field potential (LFP) recordings from bilateral STN and DBS were performed through electrode extension cables, which were externalized in the time period between electrode insertion and implantation of the subcutaneous pacemaker approximately one week after the first operation. All experiments were conducted in the morning after overnight withdrawal of dopaminergic medication, since STN beta power (13-30 Hz), which was used as feedback signal for aDBS, is more pronounced at low levels of dopamine [20].

In accordance with the declaration of Helsinki, participants gave written informed consent to participate in the study, which was approved by the local ethics committee (Oxfordshire REC A), and registered at ClinicalTrials.gov (NCT02585154).

### Method Details

#### Sample size

We conducted a sample size estimation using G^∗^Power [42] before conducting the experiment. Since there are no previous studies reporting how adaptive deep brain stimulation (aDBS) affects decision-making processes, we based our experimental task on a previous continuous DBS (cDBS) study [19]. In this previous study cDBS diminished the effect of task difficulty (manipulated using different coherence levels in a moving dots task) on response times (RT). Accordingly, we analyzed the required sample size for the difference between cDBS and off DBS. In the study by Green et al. [19] RT difference between the coherence levels 8% and 50% (which were used in the current study) was approximately 75 ± 50 ms standard error of the mean (SEM) with stimulation and 230 ± 50 ms SEM without stimulation (when averaged across speed versus accuracy instructions). Assuming a correlation between on and off stimulation measures of 0.8 (correlation in the current study was ∼0.9), this resulted in an effect size of 1.75 and, given an alpha of 0.05 and power of 0.9, a required sample size of n = 6 for a Wilcoxon signed rank test. To allow for drop-outs and ‘small-study effects’, which posit that studies with low sample sizes often overestimate effect sizes [43], we opted to include ten patients.

#### Determining contacts for LFP recordings and electrical stimulation

First, we obtained bilateral STN LFP recordings from the implanted quadripolar macroelectrodes (model 3389, Medtronic Neurological Division, Minneapolis, MN, USA) during rest for ∼1 min. The four contacts were numbered from 0 to 3 with contact 0 being the most ventral and contact 3 being the most dorsal. LFPs were recorded in a bipolar montage between contact 0 and 2 (ventral bipolar) as well as between contact 1 and 3 (dorsal bipolar) using a custom-built closed-loop device (see below). Then, we computed the Fourier transform of the recorded signal using Spike2 software (Cambridge Electronic Design, Cambridge, UK) with a frequency resolution of 1Hz and visually inspected the resulting frequency spectra between 1 and 50 Hz. For each patient, the individual peak in the beta frequency band was noted and its power compared between the ventral and dorsal bipolar recordings. The bipolar channel with the strongest beta power was used for subsequent recordings and online analysis of beta power. In two patients, octopolar non-directional macroelectrodes (model DB-2202, Boston Scientific, Marlborough, MA, USA) were implanted. In these patients, we first recorded from all eight contacts using a TMSi porti (TMS International, Enschede, the Netherlands) and then connected the four consecutive contacts showing strongest beta power to the closed-loop device. The following steps were identical for quadripolar and octopolar electrodes.

#### Adaptive DBS

The system for aDBS has been validated and described in detail previously [20, 24, 44]. In short, LFPs were recorded with a band-pass filter between 3-37 Hz and amplified using common mode rejection with a custom-built device, and analog-to-digital converted using a 1401 data acquisition unit (Cambridge Electronic Design, Cambridge, UK). This approach enabled us to record STN power in the beta band, however we were not able to record low frequency oscillations around the theta-frequency (∼2-8 Hz), which also have been related to decision threshold adjustments. Signal processing was carried out using Spike2 software on a portable computer. Based on the rest recordings we defined the individual beta peak frequency (see above) and filtered the signal around this peak (frequency band shown for each patient in Table S1). This digitally filtered signal was then rectified and smoothed using a 400 ms moving window. Based on previous studies [20, 24, 44] we set the threshold so that stimulation was triggered ∼50% of the time. To mitigate paraesthesias induced by abrupt stimulation onset, stimulation was ramped up and down for 250 ms (see Figure S1A). A 500 ms lockout period after the trigger-off signal was used to prevent any putative trigger-off artifacts from triggering stimulation. It is important to note that stimulation was triggered ∼50% of the time, but that stimulation at the clinically effective voltage was applied considerably less frequently, since stimulation always ramped up before reaching the clinically effective voltage. The active contact (cathode) for applying stimulation was localized in between the contacts used for LFP recordings (i.e., contact 1 when recording from 0-2 or contact 2 when recording from 1-3) enabling common mode rejection to minimize the stimulation artifact. A self-adhesive electrode (Pals, Nidd Valley Medical, Bordon, UK) attached at the lower neck (∼C7) served as reference for pseudo-monopolar stimulation.

We determined the clinically effective voltage of aDBS by slowly increasing the voltage in steps of 0.5 V until a clear benefit in rigidity and / or bradykinesia was observed or side effects (most notably paresthesia) became apparent. The voltage that yielded clinical benefit without evoking side effects was noted and used throughout the experiment. Stimulation voltage and timing was optimized and controlled independently for both hemispheres [24, 44]. DBS pulses were charge-balanced with a pulse width of 60 μs, a 20 μs delay between the symmetrical anodal and cathodal pulse and a fixed frequency of 130 Hz. Of note, the stimulation settings were optimized for aDBS and due to time limitations and to avoid patient fatigue (the experiment lasted ∼3 h), we used the same parameters for cDBS.

#### Experimental task

We used a modified moving dots task validated in a previous study [12]; see Figure 1A. The task was presented on a MacBook Pro (OS X Yosemite, version 10.10.3, 13.3 inch Retina display, 60 Hz refresh rate) using PsychoPy v1.8 [45]. The display was viewed from a comfortable distance while allowing the subjects to interact with the keyboard. At the beginning of each trial a text cue indicated whether participants should respond as quickly (“Fast!”) or as accurately as possible (“Accurate!”). The duration of this cue was randomly jittered between 0.75 and 1.25 s with an average duration of 1 s. Then, a cloud of 200 randomly moving white dots was presented on a black background. The diameter of the cloud was 14 cm and dot size was 10 pixels. Each dot moved in a straight line at a rate of 0.14 mm per frame for 20 frames before moving to another part of the cloud where it moved in a new direction chosen pseudorandomly between −180° and 180°. While some of the dots were moving randomly, the remaining dots moved coherently in one direction, which made the cloud of dots appear to move to the left or right. Participants were instructed to press a key with their right index finger (“/” on the right side of the laptop keyboard) if they perceived that the cloud was moving to the right and to press a key with their left index finger (“z” on the left side of the laptop keyboard) when they perceived a leftward movement. Between responses both index fingers rested on the respective keys. The percentage of dots moving coherently in one direction was either 50% (high coherence) or 8% (low coherence). These two cues were pseudorandomly presented with equal probability so that participants could not predict whether the next trial would contain dot movements with high or low coherence. The trial was terminated by a response or after a 3 s deadline in case participants did not respond followed by immediate visual feedback, which was shown for 500 ms. During accuracy instructions “incorrect” was shown as feedback both for errors of commission and errors of omission, while “correct” was shown for all correct trials. During speed instructions “in time” was shown for all responses within the 3 s window, while “too slow” was shown if patients did not respond within the 3 s deadline. Cue onset and responses triggered a TTL pulse that was sent to Spike2 through a labjack u3 system (Labjack Corporation, Lakewood, CO, USA) in order to synchronize task events with the LFP recordings and stimulation pulses. Similar to previous studies of speed-accuracy adjustments in PD, we did not impose a more restricted time window for responding during speed instruction [19, 46], since motor function varies considerably between PD patients. While trials with different coherence levels were randomly interspersed, accuracy and speed trials alternated in blocks of 20 trials [19]. These blocks were repeated 6 times each resulting in 240 trials for the whole test (Figure 1B), which lasted approximately 10 min. Before commencement of the experimental recordings patients could practice the task for as long as they wished (usually approx. 40 trials).

#### Order of sessions and clinical evaluation of DBS

The patients performed the task three times: off DBS, during cDBS and during aDBS. After adjustments of the stimulator settings in each condition (off DBS, cDBS and aDBS) patients rested for 10 min and then performed the experimental task. The order of sessions was pseudorandomized and counterbalanced across subjects (since seven subjects completed the task, the order “aDBS-off DBS-cDBS” was used twice; the other five possible combinations were used once) in order to control for changes in motivation and arousal state. After patients completed the task in each condition, the Unified Parkinson’s Disease Rating Scale (MDS-UPDRS), part III was assessed and videotaped to allow blinded evaluations. After completion of the study, which lasted ∼3 h in total, patients received their usual dopaminergic treatment and returned to the ward.

The UPDRS-III ratings were conducted offline by a movement disorder specialist blinded to the type of stimulation except for rigidity, which was rated by a medically trained researcher during the experiment.

### Quantification and Statistical Analysis

#### Analysis of behavioral data

Prior to statistical analyses, trials without responses (errors of omission) or RT < 0.25 s were excluded (combined 0.02% of all trials). Furthermore, patient #4 had to abort the experiment after 60 trials in the third condition (off DBS) due to fatigue leaving 540 of 720 trials available for analyses for this patient. Due to the low sample size, we used non-parametric tests for all analyses. First, we tested the effect of coherence (high versus low) and instruction (speed versus accuracy) on RT and accuracy rates off DBS by comparing the observed effects (n = 7) against 0. Then we tested whether these effects were affected by DBS irrespective of the type of stimulation (i.e., both aDBS and cDBS) and whether this differed between aDBS and cDBS. For this, we used the % change, e.g., (RT_lowcoherence_-RT_highcoherence_) / RT_highcoherence_, to control for overall changes in RT. We also show corresponding results when using the absolute change (ms), which were highly similar to % change, in Table S2. Of note, despite using a fixed lockout period after triggers were turned off (see “Adaptive DBS”) aDBS was triggered by an unusually prolonged trigger-off artifact in one patient. The artifact was elicited when stimulation was turned off and had spectral properties in the beta-range, which triggered stimulation (Figure S5). Therefore, this patient (patient #7) had to be excluded from the latter analysis leaving n = 6 for direct comparison of aDBS and cDBS. All comparisons were conducted using Wilcoxon signed rank tests in MATLAB (R2015a, The MathWorks, Natick, MA, USA) with an alpha of 0.05. Z-values thus refer to Wilcoxon’s z (approximation).

#### Analysis of stimulation patterns during aDBS

During aDBS, stimulation was turned on and off depending on the level of beta power. Since beta power is also modulated by the experimental task employed in the current study, with a decrease in beta power shortly after onset of the moving dots cue and a decrease in beta power around the time of the response (see [12] for more details), we assessed whether the stimulation patterns during aDBS also changed dynamically during the task. To this end we computed the % time in which stimulation was turned on using 100 ms windows, which were shifted by 10 ms from 1 s before until 1.5 s after the cue and response, respectively, and averaged the resulting values across trials. For example, a value of 25% indicates that during the respective 100 ms window, stimulation was turned on for 25ms on average. Importantly, we considered the clinically effective voltage (see “Adaptive DBS”) as stimulation ON so that the 250 ms in which stimulation was ramped up was considered as stimulation OFF. Therefore, the time in which clinical effective stimulation was applied was considerably shorter (∼20% on average) than the time in which the trigger was turned on (∼50%). This is illustrated in Figures S1A and S1B. To derive a ‘hypothetical’ pattern of aDBS in which DBS follows beta power modulations, but no actual stimulation is applied, we used the off DBS condition and computed when stimulation would have been triggered. In more detail, we set a surrogate trigger to ON whenever beta power crossed a ∼50% threshold (we used the exact % which was used during aDBS for each patient, since the % trigger ON varied slightly across patients) and to OFF when beta power fell below this threshold. Then, the first 250 ms of trigger ON were removed (set to OFF) to account for ramping up of stimulation and to derive a ‘surrogate stimulation’ off DBS. In other words, this surrogate stimulation indicates when stimulation would have been triggered in the off DBS condition, but no actual stimulation was delivered (Figure S1C). This control condition was important in order to test whether the observed timing-specific effects of aDBS on behavior and STN activity (see below) were related to actual stimulation or rather to fluctuations in beta power (which triggered stimulation).

After having established task effects of aDBS stimulation patterns, we asked how these temporal patterns of stimulation affected patients’ behavior. During aDBS, the average duration of stimulation pulses was ∼200 ms excluding ramping. Thus, whether or not stimulation was applied varied during the task-related time windows and over trials, which is illustrated in Figure 3A. In this example, in time window 1 (blue rectangle in the figure) starting 50 ms after the cue and lasting 100 ms, stimulation was applied in trial 1, 2 and 4, but not 3, while in later time windows (green and red rectangles) stimulation was applied only in trials 1, 3 and 4 and 1-3, respectively. For each trial, we noted for each 100 ms time window whether stimulation was applied or not (irrespective of the duration of stimulation) and then shifted the time window by 10 ms from onset of the moving dots cue to 1 s after the cue and 1 s before the response until the response. Thus this analysis indicated on a trial-by-trial basis whether stimulation was applied in different task-related time windows. While the number of trials in which stimulation was applied varied to some extent depending on the time window (analogously to the time-of-stimulation analysis in Figure S1B), its range was limited to 35%–45% on average across all time windows (right panels in Figure 3A). Thus, this analysis allowed us to assess behavioral effects of stimulation (i.e., trials with stimulation versus trials without stimulation) within the aDBS condition for distinct task-related time windows. We computed the effect of coherence (low versus high coherence) and instruction (accuracy versus speed) and then subtracted trials without stimulation from trials with stimulation, i.e., for the effect of coherence we used (RT_lowcoherence_stim_-RT_highcoherence_stim_) - (RT_lowcoherence_nostim_-RT_highcoherence_nostim_) and for the effect of instruction we computed (RT_Accuracy_stim_-RT_Speed_stim_) - (RT_Accuracy_nostim_-RT_Speed_nostim_). Thus, for effects of coherence, negative values indicate that patients’ slowed down less depending on task difficulty, while for the effect of instructions negative values indicate that patients’ reduced RT less after speed instructions during stimulation compared to no stimulation. Of note, in this analysis all seven patients could be included (patient #7 was excluded in the previous behavioral DBS analysis), since here we were only interested when stimulation was applied, not whether it was triggered by beta power. To correct for the multitude of tests conducted in this analysis we applied cluster-based permutation tests. First, values at each time point were z-scored and thresholded at alpha = 0.05 correcting for multiple comparisons by computing norminv(1 – 0.05/4) = 2.24, where norminv is the normal inverse cumulative distribution function. Thus, this test corrected for the four tests conducted (effect of coherence and effect of instruction for cue- and response-related time windows). Next, the resulting clusters, which consisted of all time points that exceed this threshold, were compared against the probability of clusters occurring by chance by randomly shuffling between condition labels (stimulation versus no stimulation) using 1000 permutations. Only clusters in the observed data that were larger than 95% of the distribution of clusters obtained in the permutation analysis were considered significant thereby correcting for the number of time points tested in each analysis.

Importantly, this analysis contained a possible confound, since aDBS stimulation was triggered by beta power. Thus, any effects observed when stimulation was turned on could simply be related to beta power being high during that time window (or more precisely several hundred ms before the time window, since stimulation ramped up for 250 ms). To control for this, we used the ‘surrogate stimulation’ off DBS described above and carried out the identical analysis as before. That is, we again assessed whether there were any time window specific effects on coherence and instruction for cue- and response-aligned time windows, but now we used the surrogate stimulation off DBS instead of real stimulation during aDBS. Both analyses were related to time-variant changes in beta power, but only during aDBS real stimulation was applied.

#### Drift Diffusion Model

In the drift diffusion model (DDM) framework, perceptual decision-making between two alternatives is reflected by a continuous integration of relative sensory evidence over time until sufficient evidence has been accumulated and the choice is executed. DDM has been widely applied over the last decades and has been shown to accurately predict behavior over a range of different tasks [4]. There are three main parameters in DDM. First, the drift rate *v* reflects the rate of evidence accumulation. If a cue clearly favors one over the other choice the drift rate is high resulting in fast and accurate decisions, while ambiguous cues will lead to low drift rates and thus slow and error-prone choices. Second, the decision threshold *a* defines how much evidence is accumulated before committing to a choice. Thus, the decision threshold constitutes a decision criterion, which transforms a continuous variable (sensory evidence) into a categorical choice (option A or B). The third parameter in DDM is the non-decision time *t*, which is thought to be related to afferent delay, sensory processing and motor execution.

We applied a Bayesian hierarchical estimation of DDM (HDDM) [22], implemented in Python 2.7. The hierarchical design assumes that parameters *v*_s_, *a*_s_ and *t*_s_ from individual participant s are not completely independent, but drawn from the group distributions with means μ_v_, μ_a_, μ_t_ and standard deviations σ_v_, σ_a_, σ_t_ allowing variations from the means given sufficient evidence to overwhelm the group prior. Prior distributions of μ_v_, μ_a_, μ_t_ and σ_v_, σ_a_, σ_t_ were informed by 23 previous studies [22]. The starting parameter (bias parameter) *z* was fixed to 0.5, because leftward and rightward movements were equally likely. In a first step, we constructed a simple model in which the drift rate varied between trials with different levels of coherence, the decision threshold varied according to task instructions (speed versus accuracy) and the non-decision time was kept constant based on [12] Furthermore, decision thresholds were allowed to vary depending on task difficulty (coherence) based on evidence from empirical studies and ideal observed models [7, 8, 23].

$$a_{\mathrm{s,k}}\;=\;a_{s}\;+\;b_{1}\mathrm{Inst}r_{k}\;+\;b_{2}\mathrm{Co}h_{k}$$

(Equation 1) $$v_{\mathrm{s,k}}\;=\;v_{s}\;+\;b_{3}\mathrm{Co}h_{k}$$

where *a*_*s,k*_ and *v*_*s,k*_ are the decision threshold and the drift rate of participant *s* on trial *k*, *Instr*_*k*_ the Instruction on trial *k* (0 for accuracy, 1 for speed), *Coh*_*k*_ the coherence on trial *k* (0 for high coherence, 1 for low coherence) and *b*_*1-3*_ the estimated regression coefficients.

Rather than exploring a large model space with different combinations of interactions between experimental manipulations and model parameters, we used this simple model and then assessed model performance, i.e., the ability of the model to predict the observed data, using posterior predictive checks (see below). Markov chain Monte Carlo sampling was used for Bayesian approximation of the posterior distribution of model parameters. To ensure model convergence we inspected traces of model parameters, their autocorrelation and computed the R-hat (Gelman-Rubin) statistics [22]. To assess model performance we computed quantile probability plots [4], in which predicted (ellipses) and observed RT (crosses) for the 10, 30, 50, 70 and 90 percentile were plotted against their predicted and observed cumulative probability for each condition. Of note, the group ‘average’ in Bayesian hierarchical models refers to the group prior from which the individual parameters are drawn. Error trials were only plotted for the low coherence condition due to the paucity of errors in the high coherence condition (< 4%). Note that in these plots the probability on the x axis is the product of the quartile (e.g., 0.9) times the accuracy (e.g., 0.95). For example in Figure 4B, the probability of correct trials (blue) is lower during low coherence trials compared to high coherence trials (i.e., shifted to the left on the x axis) due to the higher amount of errors during low coherence. Thus, if the model does not properly predict accuracy rates, the spheres (predictions) would be moved to the left or right of the crosses (observed data). Thus both predictions of RT and accuracy can be inferred from the plots. To assess the generalizability of our model, we also plotted the observed cumulative probability of RT from an independent patient group performing the identical task [12]. Parameters of the model were analyzed by Bayesian hypothesis testing. For all HDDM analyses, we considered posterior probabilities ≥ 95% of the respective parameters being different than zero significant [11, 12, 13]. In other words, model parameters were significant if ≥ 95% of samples drawn from the posterior were different from zero. For comparisons between conditions, model parameters were considered significant if samples drawn from the posterior were different from the distribution they were compared to (e.g., stimulation versus no stimulation) in ≥ 95% of the iterations/samples.

After having conducted the HDDM analysis for patients off DBS, we assessed whether DBS during specific time windows altered model parameters using the behavioral data from aDBS for model fitting. We marked each trial as ‘on’ if stimulation was applied and ‘off’ if no stimulation was applied during the respective time windows. Based on the behavioral results we extended our model so that model parameters (*a*, *v* and *t*) could also be modulated by cue-locked stimulation in addition to their task-related modulations.

$$a_{\mathrm{s,k}}\;=\;a_{s}+\;b_{1}\mathrm{Inst}r_{k}\;+\;b_{2}\mathrm{Co}h_{k}\;+\;b_{3}\mathrm{Sti}m_{\mathrm{s,k}}+\;b_{4}\mathrm{Inst}r_{k}\;*\;\mathrm{Sti}m_{\mathrm{s,k}}\;+\;b_{5}\mathrm{Co}h_{k}\;*\mathrm{Sti}m_{\mathrm{s,k}}$$

$$v_{\mathrm{s,k}}=\;v_{s}\;+\;b_{6}\mathrm{Co}h_{k}+\;b_{7}\mathrm{Sti}m_{\mathrm{s,k}}\;+\;b_{8}\mathrm{Co}h_{k}\;*\;\mathrm{Sti}m_{\mathrm{s,k}}$$

$$t_{\mathrm{s,k}}\;=\;t_{s}\;+\;b_{9}\mathrm{Sti}m_{\mathrm{s,k}}$$

where *t*_*s,k*_ is the non-decision time of participants *s* on trial *k* and *Stim*_*s,k*_ the presence of cue-locked stimulation for participants *s* on trial *k* (0 for Off, 1 for On).

We then assessed whether these effects were significant using Bayesian hypothesis testing (see above) and explored significant stimulation effects (*Coh^∗^Stim*, see results) using post hoc tests for the significant interaction, i.e.

$$a_{\mathrm{s,k}}\;=\;a_{s}\;+\;b_{1}\mathrm{Inst}r_{k}\;+\;b_{2}\mathrm{Coh}\text{\_}\mathrm{Sti}m_{\mathrm{s,k}}\;+\;b_{3}\mathrm{Coh}\text{\_}\mathrm{noSti}m_{\mathrm{s,k}}+\;b_{4}\mathrm{Sti}m_{\mathrm{s,k}}$$

$$v_{\mathrm{s,k}}\;=\;v_{s}\;+\;b_{5}\mathrm{Co}h_{k}$$

where *Coh_Stim*_*s,k*_ is the coherence during trial *k* on stimulation of subject *s* and *Coh_noStim*_*s,k*_ is the coherence during trial *k* off stimulation of subject *s.*

Finally, we conducted the same analysis for cDBS, where Stim refers to the cDBS condition and noStim to off DBS.

#### Analysis of electrophysiological data and HDDM regression analyses

We analyzed the recorded STN LFP using a hypothesis-driven approach. First, we assessed whether there were correlations between the early (150-400 ms) cue-induced decrease in beta power off DBS and trial-by-trial adjustments in decision thresholds as observed in our previous study [12] for replication purposes and to validate that STN beta power was related to changes in decision thresholds. Second, we analyzed STN beta power during high and low coherence trials following cue onset, since here DBS-related changes in patients’ ability to slow down responses were observed (see results).

For LFP analysis, we analyzed the analog filtered (3-37 Hz) bipolar signals using MATLAB. Trials with artifacts were discarded after visual inspection. After removal of trials based on behavioral data (see above) and artifacts in the electrophysiological data on average 622 trials (86.4%) remained per patient resulting in 4354 trials combined. Data were down-sampled to 200 Hz and high-pass filtered at 1 Hz. Power of LFPs were computed using the continuous wavelet transform with 2 cycles per frequency for the lowest considered frequency (3 Hz) which linearly increased to 5 cycles per frequency for the highest considered frequency (30 Hz) in 1 Hz steps. Power of each frequency was normalized to the mean signal of that frequency across the whole experiment. Since we selected the channel showing the stronger beta power for LFP recordings (see “Adaptive DBS”) and stimulation triggers induced a low-frequency artifact, we only analyzed beta power between 13-30 Hz (for individual beta peak frequencies see Table S1). The resulting traces were aligned to the onset of the moving dots and averaged across hemispheres resulting in one STN channel per patient.

First, we assessed task-related changes in STN beta power off DBS. We computed changes in beta power averaged across all conditions, separately for speed and accuracy instructions and separately for low and high coherence trials. In order to take into account RT differences between conditions we computed the change in beta power over time until the response was executed, i.e., the time series of each trial was capped at the time of the response. We then averaged these time series across trials from 100 ms before dots onset until the point in time when 50% of trials (for the respective conditions) contributed to the average. Thereby we ensured the inclusion of at least 50% of trials at all considered time points for all considered conditions [12]. To compare beta power changes after speed versus accuracy instructions, we computed the differences in beta power from 100-150 ms postcue (highest beta power after the cue) to the individual beta trough ∼400 ms (±100 ms) after the cue (see Figure 5A) based on [12]. Then, we compared this early cue-induced beta decrease between speed and accuracy conditions, as well as between low and high coherence, using Wilcoxon signed rank tests. In a next step, we then assessed whether STN activity changes were related to adjustments in decision thresholds on a trial-by-trial basis. Identical to our previous work [12] we computed the cue-induced change in beta power (using the individual beta frequency peaks) for each trial as detailed above. Then, values were z-scored by subtracting the mean and dividing by the standard deviation for each subject. The resulting values were then entered into the HDDM and regressed against estimates of thresholds at each trial during model estimation. In other words the regression coefficients between STN activity and decision thresholds were estimated within the same model, which was used to estimate the decision-making parameters themselves. Specifically, on a given trial the threshold *a* was defined by:

$$a_{\mathrm{s,k}}\;=\;a_{s}\;+\;b_{1}\mathrm{Inst}r_{k}\;+\;b_{2}\mathrm{Co}h_{k}\;+\;b3\mathrm{Betadecreas}e_{\mathrm{s,k}}$$

where *Betadecrease* is the cue-induced beta decrease (continuous variable) of participant *s* on trial *k*.

Thus, this model specifically tested whether trial-by-trial variations in the cue-induced beta decrease predicted changes in decision threshold (irrespective of trial type) based on our previous observations [12]. As before, the drift rate was assumed to depend on coherence (analogously to Equation 1). Posteriors of regression coefficients for trial-wise regressors were estimated only at the group level to address potential collinearity among model parameters, for regularizing parameter estimates and to prevent parameter explosion [11, 22]. Statistical inference on regression coefficients was based on the distribution of the posterior probability densities (see above).

In a second step, we analyzed beta power in low versus high coherence trials. While beta power in high coherence trials further decreased after the early cue-induced beta decrease presumably due to the low RTs in this condition, beta power showed a relative increase from ∼500-800 ms after the cue in low coherence trials (see Figure 5A). Since this change in beta frequency occurred immediately after the time period in which stimulation affected patients’ ability to slow down (400-500 ms postcue, see results), we analyzed whether STN beta power in this time period also reflected changes in decision thresholds. Analogously to the HDDM analysis using the cue-induced beta decrease described above, we entered single trial z-scored estimates of beta power from 500-800 ms into the HDDM and tested whether trial-by-trial fluctuations in beta power correlated with changes in decision thresholds. Of note, this was conducted for low and high coherence trials separately, since the relative beta increase was only observed in low coherence trials and the response (with a concomitant decrease in beta power) fell into the 500-800 ms window in high coherence trials. Thus, the threshold was defined by:

(Equation 2) $$a_{\mathrm{s,k}}\;=\;a_{s}\;+\;b_{1}\mathrm{Inst}r_{k}\;+\;b_{2}\mathrm{Co}h_{k}\;+\;b_{3}\mathrm{Beta}\text{\_}LC_{\mathrm{s,k}}\;+\;b_{4}\mathrm{Beta}\text{\_}HC_{\mathrm{s,k}}$$

where *Beta is* the beta power from 500-800 ms postcue for low coherence (*LC*) and high coherence (*HC*) trials *k* of participant *s*.

To make sure that any correlations were not driven by trials in which the response fell into the 500-800 ms window, we repeated this HDDM analysis excluding all low coherence trials with RT < 800 ms from the regression analysis. Furthermore, we tested the reproducibility of the results by conducting the same analysis (excluding RT < 800 ms) using data from a group of 11 independent PD patients [12].

Finally, we analyzed how stimulation altered task-related modulation of STN beta power. During aDBS, stimulation was triggered by beta power and therefore beta power was necessarily high in stimulation trials a few hundred ms (stimulation was ramped for 250 ms) before the respective time window. To control for this when comparing stimulation and no stimulation trials, we compared trials in which stimulation was applied 400-500 ms postcue during aDBS to ‘surrogate’ stimulation trials off DBS and during cDBS (see above). We focused our analysis on the time window 500-800 ms after the cue in low coherence trials based on the results off DBS (see above). Beta power in this time window was compared between off DBS and DBS (irrespective of the type of stimulation) and between aDBS and cDBS using Wilcoxon signed rank tests. Then, we entered single trial estimates of beta power 500-800 ms postcue into the HDDM for cDBS and aDBS analogously to the HDDM analysis with post-cue beta power off DBS (Equation 2). Of note, for this analysis aDBS was not further subdivided into stimulation versus no stimulation 400-500 ms postcue, since only low coherence trials could be used for regression analyses, which would leave less than 20% of trials when subdividing the data further. As above, statistical inferences on regression coefficients were based on the distribution of the posterior probability densities.

### Data and Software Availability

Behavioral and neurophysiological data related to the project is freely available at https://ora.ox.ac.uk/objects/uuid:0cdf0eda-3a1d-4b66-8edd-0f51d824f6cc.

## Supplemental Information

Document S1. Figures S1–S5 and Tables S1 and S2

Document S2. Article plus Supplemental Information
