# Supplementary material for: Mechanisms Underlying Decision-Making as Revealed by Deep-Brain Stimulation in Patients with Parkinson’s Disease
Source: Curr Biol. 2018 Apr 23;28(8):1169–1178.e6. doi: 10.1016/j.cub.2018.02.057 (PMC5912902; doi:10.1016/j.cub.2018.02.057)
Supplement: Document S1. Figures S1–S5 and Tables S1 and S2 [file mmc1.pdf]

Current Biology, Volume *28*

Supplemental Information

Mechanisms Underlying Decision-Making as Revealed by Deep-Brain Stimulation in Patients with Parkinson's Disease

Damian M. Herz, Simon Little, David J. Pedrosa, Gerd Tinkhauser, Binith Cheeran, Tom Foltynie, Rafal Bogacz, and Peter Brown

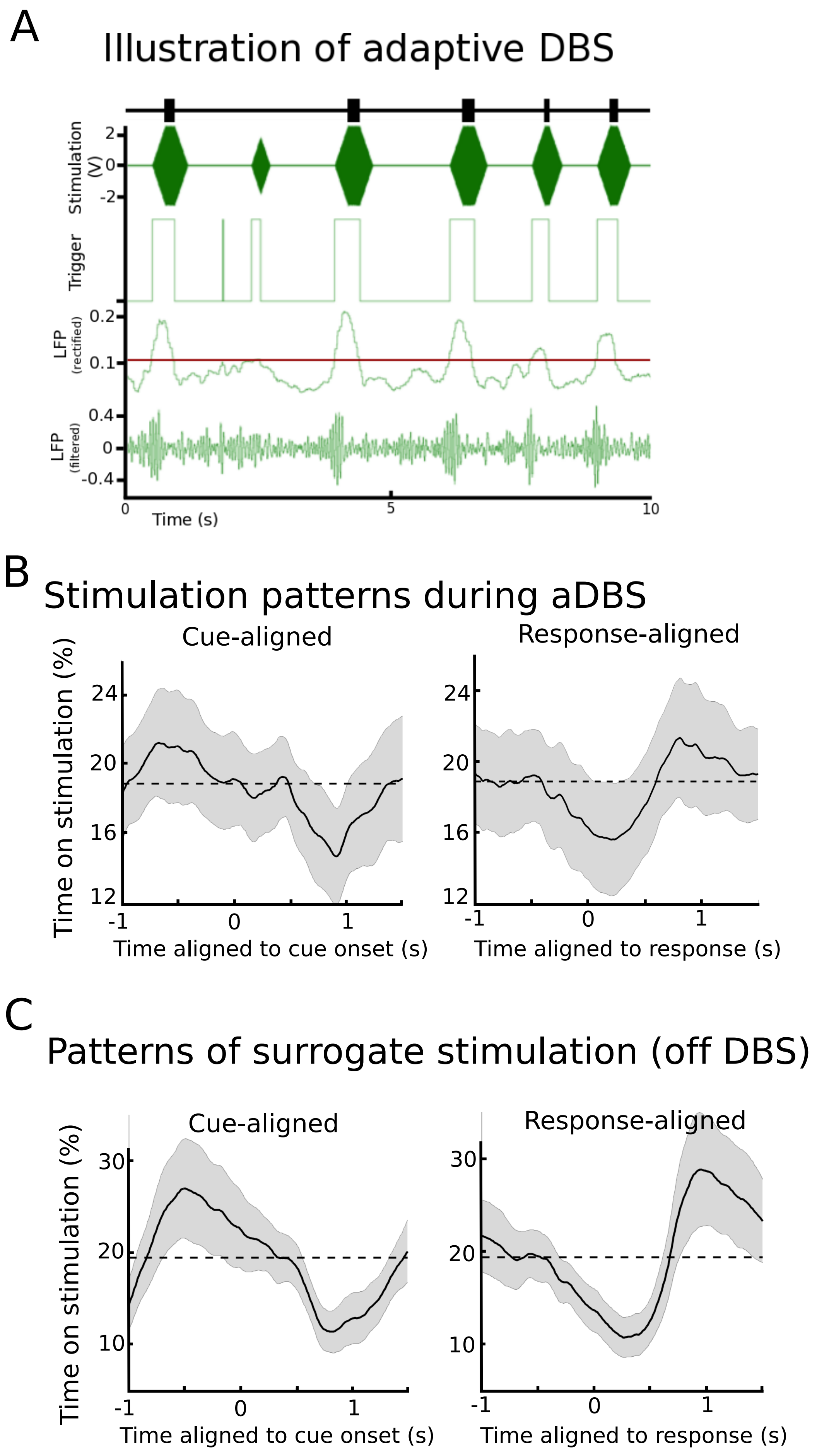
A
Illustration of adaptive DBS
Stimulation (V)
2
0
-2
Trigger
LFP (rectified)
0.2
0.1
LFP (filtered)
0.4
0
-0.4
0
5
10
Time (s)
B
Stimulation patterns during aDBS
Cue-aligned
Response-aligned
Time on stimulation (%)
24
20
16
12
-1
0
1
Time aligned to cue onset (s)
Time aligned to response (s)
C
Patterns of surrogate stimulation (off DBS)
Cue-aligned
Response-aligned
Time on stimulation (%)
30
20
10
-1
0
1
Time aligned to cue onset (s)
Time aligned to response (s)

Figure S1. Adaptive DBS. Related to figure 1.

A. Example of adaptive DBS from patient #1. The lowest trace shows beta power that is digitally-filtered around the patients' individual beta peak frequency. The second trace from the bottom shows the rectified beta activity and illustrates the threshold (red line), which was set at the median of beta power. Whenever, beta power crossed this threshold stimulation was triggered and ramped up. The uppermost trace (black) illustrates time points at which stimulation was delivered at the clinically effective voltage, i.e. the same as the trace below ('Stimulation') but without the ramping. B. Time on stimulation (ToS) for cue- and response-aligned time windows (100 ms windows shifted by 10 ms). E.g. 20% ToS indicates that during the given 100 ms time window, stimulation was applied for 20 ms on average across trials. Shaded areas represent SEM across patients. ToS follows task-related beta changes with low ToS several 100 ms after cue onset and around the response. Note that the change in ToS is lagging behind the changes in beta power due to the moving window used for online analysis of beta power and the 250 ms ramping. C. Same as B, but for off DBS. Here a 'hypothetical' threshold was set offline corresponding to the threshold set for aDBS for each individual patient. This analysis yields a 'surrogate stimulation' used for controlling for effects of fluctuations in beta power compared to analysis of adaptive stimulation effects. During both 'stimulation' was dependent on changes in beta power, but only during aDBS was real stimulation applied. Note, that surrogate stimulation off DBS follows task-related changes in beta more closely than aDBS presumably since no stimulation is applied (-the latter is known to decrease beta power).

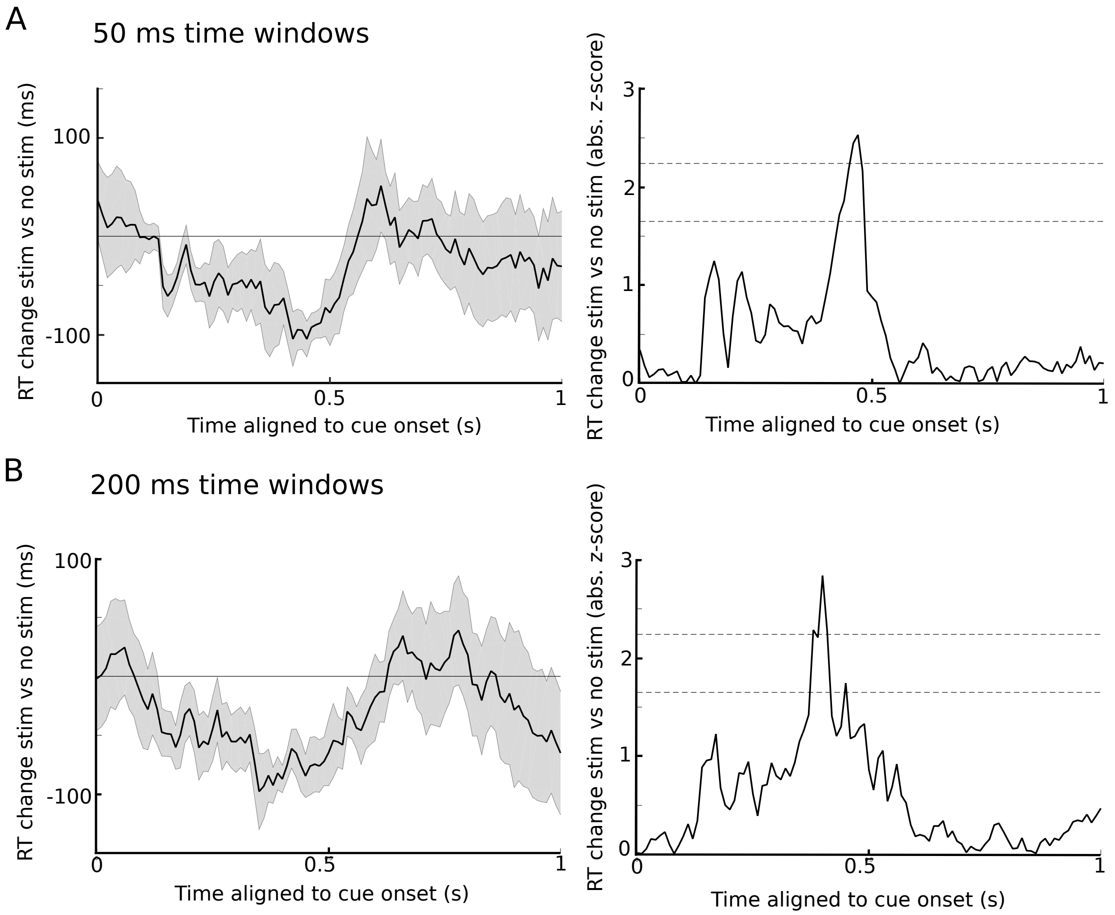

Figure S2. Time window analysis of aDBS effects on RT increase in low vs. high coherence trials with windows of different sizes. Related to figure 3. The effect reported in figure 3b (100 ms windows) is here shown using windows of 50 ms (a) and 200 ms (b). Mean ± SEM in ms is shown in the left panels, absolute z-scores (mean / standard deviation) in the right panels. As in figure 3b the horizontal lines in the right panels refer to z = 1.65 and z = 2.24 (significance threshold).

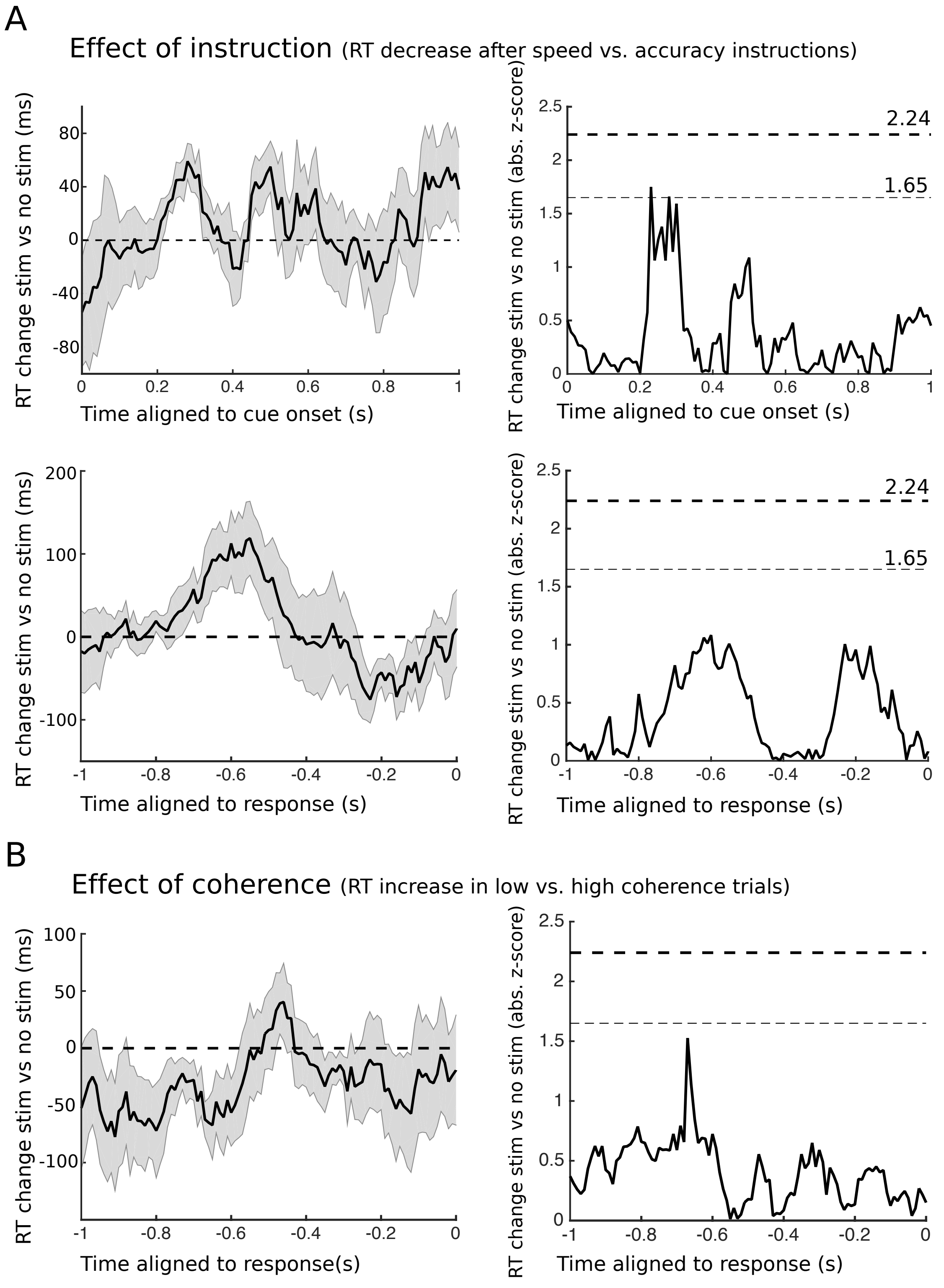
A
Effect of instruction (RT decrease after speed vs. accuracy instructions)
RT change stim vs no stim (ms)
80
40
0
-40
-80
0
0.2
0.4
0.6
0.8
1
Time aligned to cue onset (s)
RT change stim vs no stim (abs. z-score)
2.5
2
1.5
1
0.5
2.24
1.65
Time aligned to cue onset (s)
RT change stim vs no stim (ms)
200
100
-100
-1
-0.8
-0.6
-0.4
-0.2
Time aligned to response (s)
RT change stim vs no stim (abs. z-score)
2.24
1.65
Time aligned to response (s)
B
Effect of coherence (RT increase in low vs. high coherence trials)
RT change stim vs no stim (ms)
100
50
-50
-100
Time aligned to response(s)
RT change stim vs no stim (abs. z-score)
Time aligned to response (s)

Figure S3. Time window analysis of aDBS effects on behavior. Related to figure 3.

A. Changes in effects of instruction (speeding of RT after speed compared to accuracy instructions) during stimulation vs. no stimulation trials. Positive values indicate that patients sped up more after stimulation compared to no stimulation. In the upper pair of panels, analysis was conducted for 100 ms windows shifted by 10 ms from onset of the moving dots cue until 1 s after the cue. Mean ± SEM in ms is shown in the left panel, z-scores (mean / standard deviation) in the right panel. The statistical threshold for each time window was set to $z=2.24$ to correct for the four separate tests and correction for multiple time windows was conducted using cluster-based permutation tests. An uncorrected threshold of $z = 1.65$ is shown for illustration purposes. In the lower pair of panels, changes in the effect of instruction is shown for windows aligned to the response. B. Same as lower panel of A, but for the effect of coherence. Here, negative values indicate that patients' slowed down less after simulation vs. no stimulation. There were no significant clusters in any of the three analyses shown here.

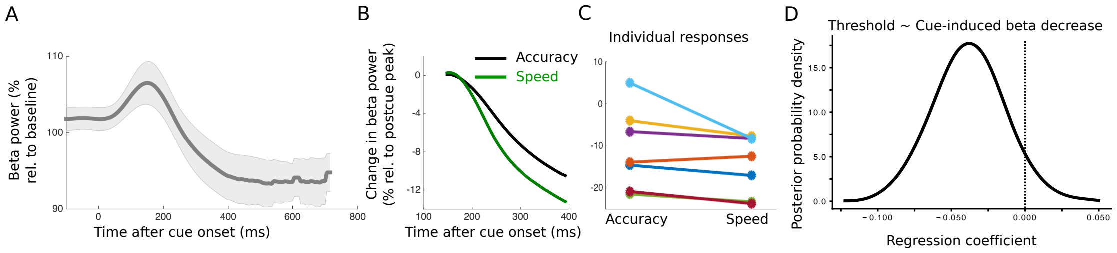

Figure S4. Cue-induced changes in beta power after speed vs. accuracy instructions. Related to figure 5.

A. When averaged across all conditions, beta power showed a steep decrease after the onset of moving dots from ~150 ms to ~400 ms postcue. B & C. This decrease was steeper after speed compared to accuracy instructions (z = 2.2, P = 0.028, Wilcoxon signed rank test). D: Trial-by-trial changes in this early (i.e. 150-400 ms postcue) beta-decrease predicted decreased decision thresholds (96% posterior probability). Note that regression coefficient is not standardized.

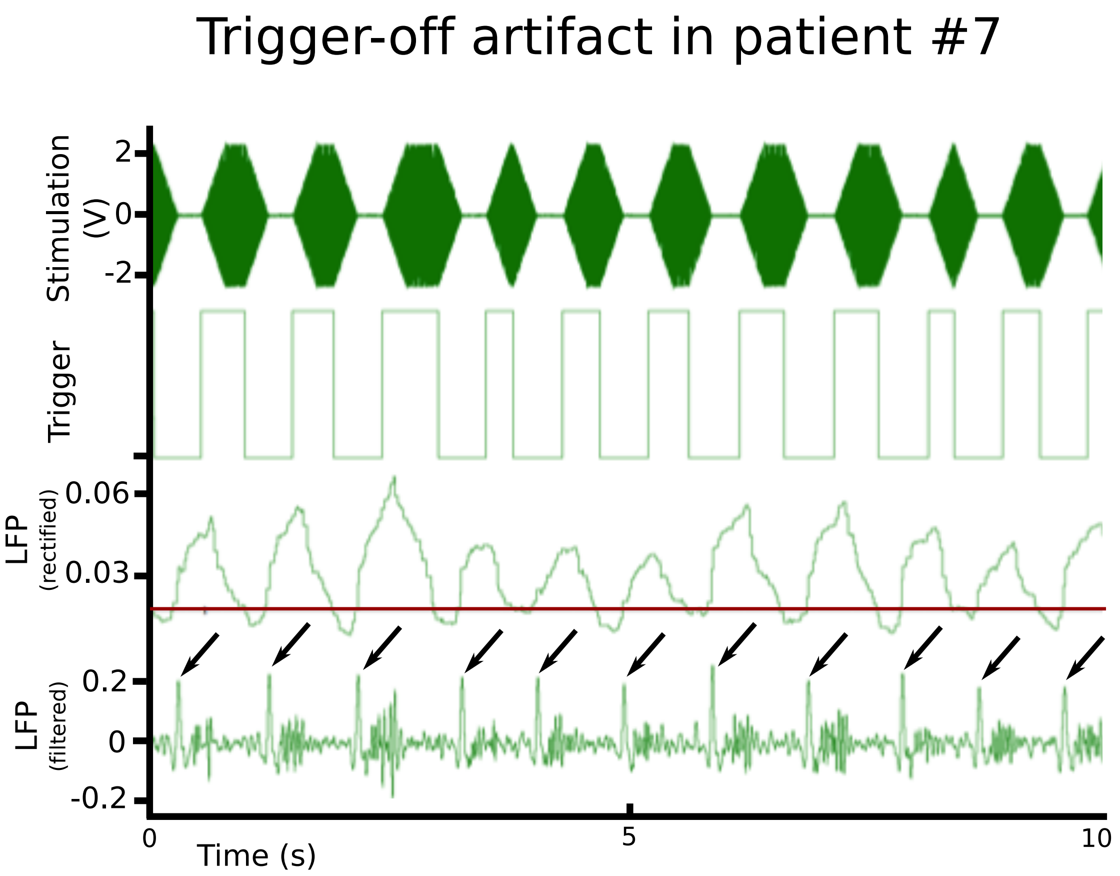

Figure S5. Trigger-off artifact in patient #7. Related to figure 1.

In this patient a large, delayed artifact (indicated by black arrows) was elicited each time stimulation had been ramped down. It disappeared when stimulation was turned off (off DBS). To account for low frequency artefacts we used a high pass filter at 3 Hz (RC filter attenuating power by 50% at 3Hz (-3dB) with a 20dB per decade roll-off). However, since this artifact had spectral properties in the beta band it was not attenuated by this filter and triggered stimulation, so that aDBS was not trigged by (endogenous) fluctuation in beta power but by stimulation itself. Therefore, this patient had to be excluded from analyses comparing behavior during aDBS vs. cDBS and off DBS. However, he could be included in the aDBS stimulation pattern analyses, since here our interest was whether stimulation had any timing-specific effects, but not if it was triggered by beta activity.

Patient	Age	UPDRS-III OFF/ON levodopa	Disease duration	First symptom	Reason for surgery	Medication (mg / day)	DBS lead	Surgical Centre	Beta filter	Stim L/R
1	50	37/17	4	Tremor	Motor fluctuations	Levodopa 500<br>Entacapone 1000<br>Pramipexole 0.54<br>Rasagiline 1	Medtronic 3389™	London	18 ±3	2.6D<br>2.7D
2	54	61/32	8	Tremor	Motor fluctuations	Levodopa 300<br>Pramipexole 1.05	Boston Scientific DB-2202™	Oxford	29 ±3	3.1V<br>3.1V
3	61	52/17	8	Tremor	Tremor	Levodopa 850<br>Entacapone 1000<br>Rasagiline 1	Medtronic 3389™	Oxford	18 ±3	3V<br>2.9D
4	53	49/9	13	Stiffness	Gait difficulties	Levodopa 600<br>Pramipexole 2.1<br>Selegiline 5<br>Amantadine 100	Medtronic 3389™	London	16 ±3	2.6D<br>2.7D
5	70	33/10	10	Loss of dexterity	Motor fluctuations	Levodopa 850<br>Entacapone 1000<br>Ropinirole 22<br>Rasagiline 1	Medtronic 3389™	London	Excl	Excl
6	50	40/19	4	Tremor	Dyskinesia	Levodopa 550<br>Ropinirole 6<br>Amantadine 100<br>Trihexyphenidyl 6	Medtronic 3389™	London	Excl	Excl
7	59	53/18	7	Stiffness	Dyskinesia	Levodopa 1000<br>Rotigotine 4	Boston Scientific DB-2202™	Oxford	20 ±6	2.6D<br>3.3D
8	59	53/31	6	Tremor	Dyskinesia	Levodopa 800<br>Rasagiline 1<br>Rotigotine 10<br>Amantadine 400	Medtronic 3389™	London	20 ±2	2.1V<br>1.9V
9	63	34/22	8	Masked face	Campto-cormia	Levodopa 300<br>Rasagiline 1<br>Trihexyphenidyl 6	Medtronic 3389™	London	13 ±4	2.6D<br>3.1V
10	51	46/25	12	Micrograp hia	Dyskinesia	Levodopa 500<br>Ropinirole 12<br>Amantadine 200	Boston Scientific DB-2202™	London	Excl	Excl

Table S1. Clinical specifications. Related to figure 1.

Age and disease duration are given in years. The beta filter indicates the frequency range (in Hz), which was used for the digital online filter of local field potential recordings. Under the column “Stim” the voltage (in Volts) that was applied during aDBS is listed followed by “V” in case the ventral contact (contact 1) was used as active electrode or “D” in case the dorsal contact (contact 2) was used as active electrode. “Excl” indicate that the respective patient did not complete the whole experiment due to fatigue.

Coherence	relative change (median, z, p)	absolute change (median, z, p)
Effect of DBS	-7%, z=-2.12, p=0.034	-88 ms, z=-1.88, p=0.059
aDBS vs. cDBS	-8.8 vs. 6.5%, z=0.52, p=0.6	-88 vs. -68 ms, z=-0.73, p=0.463
Timing-specific effect of aDBS	-14.7%, z=-2.37, p=0.018	-72 ms, z=-2.37, p=0.018
Timing-specific effect of aDBS vs sDBS	-14.7% vs. 0.6%, z=-2.37, p=0.018	-72 vs. -20 ms, z=-2.37, p=0.018

Instruction		
Effect of DBS	5.9%, z=1.41, p=0.158	53 ms, z=0.94, p=0.347
aDBS vs. cDBS	8.1 vs. -1.1%, z=-1.57, p=0.116	67 vs. -12ms, z=1.15, p=0.249

Table S2. Relative (%) vs. absolute (ms) change for effects of DBS on coherence (high vs. low) and instruction (speed vs. accuracy). Related to figure 2 and 3.

Statistics were conducted using Wilcoxon signed rank tests. ‘Timing-specific effect’ refers to stimulation 400-500 ms postcue during aDBS. DBS, deep brain stimulation; aDBS, adaptive deep brain stimulation; cDBS, continuous deep brain stimulation; sDBS, ‘surrogate’ deep brain stimulation (see STAR methods).
